# Exploratory Study for Probiotic Enrichment of a Sea Fennel (*Crithmum maritimum* L.) Preserve in Brine

**DOI:** 10.3390/foods11152219

**Published:** 2022-07-26

**Authors:** Antonietta Maoloni, Federica Cardinali, Vesna Milanović, Andrea Osimani, Maria Cristina Verdenelli, Maria Magdalena Coman, Lucia Aquilanti

**Affiliations:** 1 Dipartimento di Scienze Agrarie, Alimentari ed Ambientali, Università Politecnica delle Marche, Via Brecce Bianche, 60131 Ancona, Italy; a.maoloni@univpm.it (A.M.); v.milanovic@univpm.it (V.M.); a.osimani@univpm.it (A.O.); l.aquilanti@univpm.it (L.A.); 2 Synbiotec S.r.l., 62032 Camerino, Italy; cristina.verdenelli@unicam.it (M.C.V.); magda.coman@unicam.it (M.M.C.)

**Keywords:** rock samphire, *Lactiplantibacillus plantarum* IMC 509, SYNBIO^®^, artificially acidified preserve

## Abstract

Considering the increasing consumer demand for vegan and vegetarian health foods, different vegetables have been already exploited to produce non-dairy probiotic foods. In addition to being rich in bioactive compounds, sea fennel (*Crithmum maritimum* L.), also known as rock samphire, represents a valuable candidate in the production of probiotic-enriched foods, and, to the authors’ knowledge, it has not yet been explored as carrier for probiotics. Hence, the present study was aimed at evaluating the survival of a commercially available probiotic formulation, SYNBIO^®^, and *Lactiplantibacillus plantarum* IMC 509 in an artificially acidified, pasteurized sea fennel preserve in brine during a refrigerated storage of 44 days. Despite slight reductions in the microbial loads, at the end of the storage, both the probiotic formulations showed loads higher than 7.0 Log CFU g^−1^ of sea fennel or mL^−1^ of brine, above the recommended administration dose to exert beneficial health effects. Thus, acidified sea fennel sprouts in brine represent a potential vehicle for probiotics delivery to humans.

## 1. Introduction

Probiotics are defined as “Live microorganisms that, when administered in adequate amounts, confer a health benefit on the host” [1]. The consumption of probiotics dates to ancient times in the form of fermented products, and the capability of these foods in improving human health has been reported for a very long period of time, being mentioned in the Biblical scriptures and further described by Hippocrates [2]. Probiotics can exert a broad range of beneficial effects on the host by (i) interacting with the gut microbiota and exerting a metabolic effect thanks to enzymatic activities in the gut lumen; (ii) interacting with the gut mucus and the gut epithelium, improving the mucosal barrier function and immune system; and (iii) signaling to the systemic immune systems and other organ systems [3].

Most commercially available probiotic-enriched foods currently consist of dairy-based products, including fermented milk drinks and yogurt; however, given the increasing consumer demand for healthy foods capable of preventing diseases and promoting well-being beyond providing basic nutrition, the incidence of lactose intolerance and allergies against milk proteins, as well as the increasing trend towards vegetarian and vegan diets, the development of non-dairy probiotic foods represents an interesting opportunity for the functional food market [4].

Probiotic-enriched foods must contain appropriately selected probiotic strains in adequate dose to confer the intended health benefits, which corresponds to a daily intake of 10^8^–10^9^ probiotic microorganisms [4]. The viability of selected probiotic strains in a food matrix could be affected by the intrinsic characteristics of the product (pH; molecular oxygen; presence of salt, sugar, and antimicrobial compounds) and the employed processing parameters (incubation temperature, heat treatment, packaging material, and storage conditions) [5]. Hence, the monitoring of viability of probiotics in food products during the intended shelf-life is of utmost importance, considering that survival and stability of probiotics in these matrices are highly strain dependent [6].

To date, numerous vegetables have been successfully exploited as carriers for delivering of probiotics to humans, including table olives, artichokes, and cabbage [7,8]. Plant-based matrices are often rich in nutrients, fibers, vitamins, minerals, and dietary bioactive phytochemicals, most of which have a crucial role in the interactions with gut microorganisms [7]. As has been recently reviewed by the latter authors, the majority of plant-based probiotic foods to date investigated have juice or smoothie texture, while only a few research studies have dealt with solid vegetable matrices [7].

Sea fennel (*Crithmum maritimum* L.) undoubtedly represents an ideal candidate for functionalization with probiotics, offering itself functional benefits due to the high content of vitamin C, omega 3 and omega 6 fatty acids, phenolic compounds, carotenoids, etc. [9,10]. The use of sea fennel in culinary preparations dates back to ancient times. Forgotten for a long time, this highly aromatic herb has recently been rediscovered, being defined by various authors as a “cash crop” or “emerging crop” for its high economical potential [11,12] due to its application both pharmacologically and in the food industry. In Mediterranean countries, including Greece, France, Spain, and Italy, sea fennel is consumed as a fresh ingredient in salads or preserved in brine, olive oil, or aqueous solutions of wine vinegar [13].

To the authors’ knowledge, no studies have yet been carried out to evaluate the potential of sea fennel as a carrier of human probiotics. Similarly, it has not yet been industrially utilized with this specific purpose. Hence, this study was aimed at evaluating the survival and stability of a commercially available formulation of human probiotics, SYNBIO^®^, and *Lactiplantibacillus plantarum* IMC 509 during prolonged storage of a sea fennel preserve under refrigerated conditions.

## 2. Materials and Methods

### 2.1. Sea Fennel Supply and Pre-Treatment

Fresh sea fennel sprouts (approximately 1.5 Kg) were kindly supplied by Rinci S.r.l. (Castelfidardo, Ancona, Italy), a manufacturer of sea fennel preserves, in November 2020. They were transported to the laboratory under refrigerated conditions (+4 °C), washed under tap water, drained for 5 min using an industrial stainless steel vegetable strainer basket, blanched at 95 °C for 30 s by immersion in boiling water, and again drained for 5 min.

### 2.2. Probiotic Bacteria Strains

*Lactiplantibacillus plantarum* IMC 509 and SYNBIO^®^, a combination (1:1) of *Lacticaseibacillus rhamnosus* IMC 501^®^ and *Lacticaseibacillus paracasei* IMC 502^®^ [14,15], were kindly supplied by Synbiotec S.r.l. (Camerino, Macerata, Italy).

SYNBIO^®^ is a patented product commercialized by Synbiotec S.r.l. as a lyophilized powder, characterized by a cell load of 10^11^ CFU g^−1^. The combination of the two bacterial strains of SYNBIO^®^, both isolated from the intestinal tract of elderly humans, was justified by Verdenelli et al. [15] based on the results collected, where the mixture expressed higher in vitro adherence to intestinal cell line than the two single strains of lactobacilli, with beneficial effects on the bowel habits [16].

*Lactiplantibacillus plantarum* IMC 509 is a further strain isolated by Synbiotec S.r.l. from the intestinal tract of elderly humans and is well characterized for its probiotic traits [15,17,18,19].

### 2.3. Preparation of Sea Fennel Preserves

The amount of lactic acid to be added to the brine salt solution to reach an equilibrium pH of 3.80, and hence to guarantee microbial safety and stability of the sea fennel preserve, was preliminary determined as follows: 35 g of blanched sea fennel sprouts were added with 105 mL of brine containing 7.0% (*w v*^−1^) NaCl and 1.0% (*w v*^−1^) fructose) and homogenized using a blender. Aliquots of the homogenate, distributed in 15 mL tubes, were separately added with increasing percentages of food grade lactic acid to achieve an equilibrium pH of ≈3.80; pH values were assessed with a pH meter (model 300, Hanna Instruments, Padova, Italy).

Glass jars that were 150 mL in size were filled with 35 g of blanched sea fennel sprouts soaked in 105 mL of brine acidified with 0.5% (*v v*^−1^) food-grade lactic acid. Jars were sealed with steel caps, pasteurized at 95 °C for 5 min in boiling water, cooled in iced water, and stored at room temperature (18 ± 2 °C) for 4 weeks to allow the pH to equilibrate; pH values of the acidified brines were assessed prior to (t_0_) and after pasteurization (95 °C 5 min), then once a week up until the desired equilibrium pH was reached.

**Figure 1:**
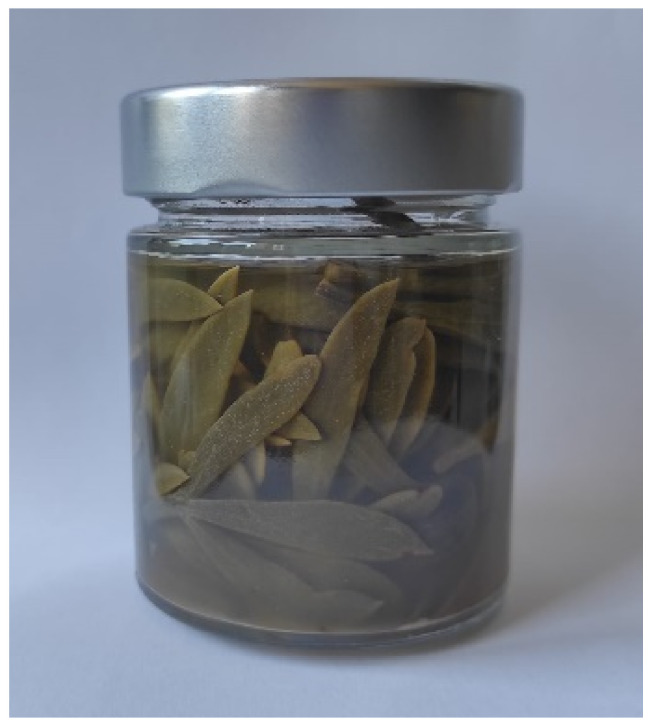
Probiotic-enriched sea fennel sprouts soaked in brine.

Once the equilibrium pH was reached, jars were separately inoculated, in triplicate, with SYNBIO^®^ or *L. plantarum* IMC 509, to reach a final load of ≈9 Log CFU mL^−1^ (Figure 1). Jars were stored at 4 ± 2 °C for 44 days.

**Figure 2:**
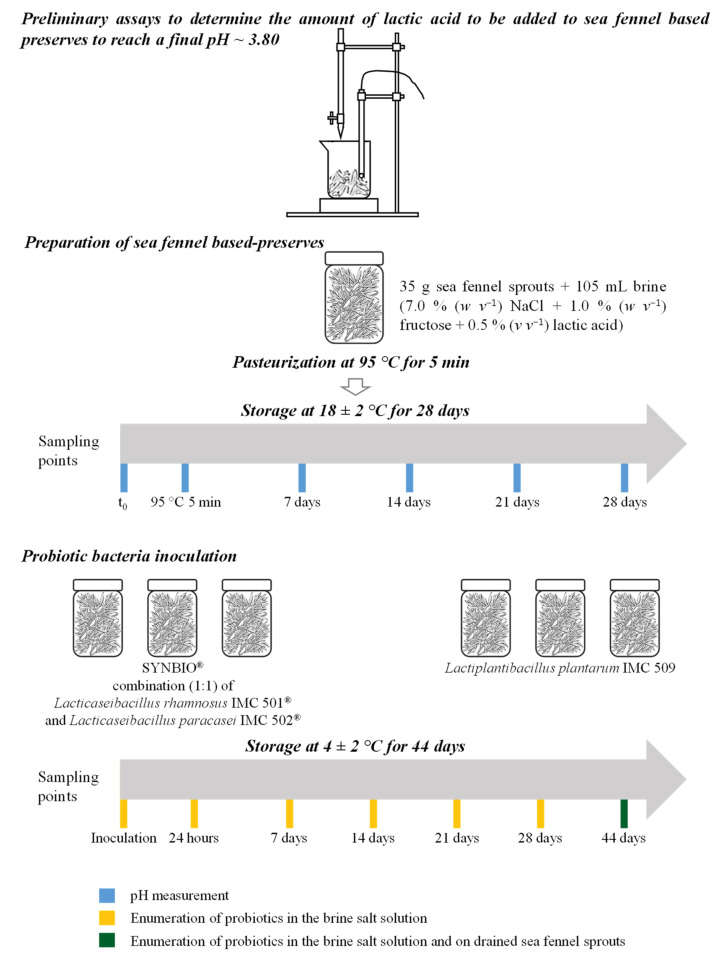
Experimental design of the study.

The experimental design of the study is depicted in Figure 2.

### 2.4. Enumeration of Probiotics in the Brine Salt Solution and on Drained Sea Fennel Sprouts

Aliquots of brine (1 mL) were aseptically collected immediately after inoculation (t_0_) and 1, 7, 14, 21, 28, and 44 days of storage under refrigeration. At the end of the monitoring period, aliquots of sea fennel sprouts (10 g) were aseptically collected from the jars with stainless-steel tweezers, drained for 2 min using a standard #8 sieve, and homogenized with sterile 0.1% (*w v*^−1^) peptone water in a Stomacher apparatus (400 Circulator, International PBI, Milan, Italy). Tenfold serial dilutions were prepared in the same diluent, and aliquots (100 µL) of each dilution were subjected to enumeration of probiotic lactobacilli on De Man, Rogosa, and Sharpe (MRS) agar (VWR, Milano, Italy) and incubated at 37 °C for 72 h. Sampling, homogenization, and preparation of serial dilutions were carried out under sterile conditions. The results of viable counting were expressed as the mean Log CFU mL^−1^ of brine or g^−1^ of sea fennel of three replicates ± standard deviation.

### 2.5. Statistical Analysis

The results overall collected were subjected to one-way analysis of variance (ANOVA) with JMP Version 11.0.0 software (SAS Institute Inc., Cary, NC, USA). Differences through multiple mean comparisons were detected performing the Tukey–Kramer honest significant difference (HSD) test (*p* ≤ 0.05).

## 3. Results and Discussion

In this exploratory study, the suitability of an artificially acidified sea fennel preserve as a carrier to deliver human probiotics was explored.

At industrial level, the manufacturing of vegetable preserves in brine involves a few main steps, namely, (i) proper rinsing of the edible portions; (ii) soaking in brine; (iii) distribution into sterilizable containers, which have to be hermetically sealed after stuffing; (iv) and thermal processing at time/temperature combinations that strictly depend on the product and the size of the container. Pasteurization is usually performed by immersion of the containers in boiling water or by exposure to steam. This thermal treatment often leads to overcooking, which might lead to detrimental effects on both the organoleptic properties (taste, flavor, texture, color, etc.) and nutritional traits of the vegetables [20].

Given these premises, the present research was aimed at developing a safe and stable unheated sea fennel preserve in brine with a high content of probiotic lactobacilli. To the authors’ knowledge, the exploitation of a sea fennel preserve as a carrier of probiotics represents an absolute novelty.

Microbial safety and stability of the preserve were guaranteed by both direct acidification of the brine salt solution, where sea fennel sprouts were soaked in, and by pasteurization at 95° for 5 min. According to “hurdle technology” theory, the combination of these treatments fully guarantees the death or growth inhibition of any potentially occurring food pathogen [21].

**Figure 3:**
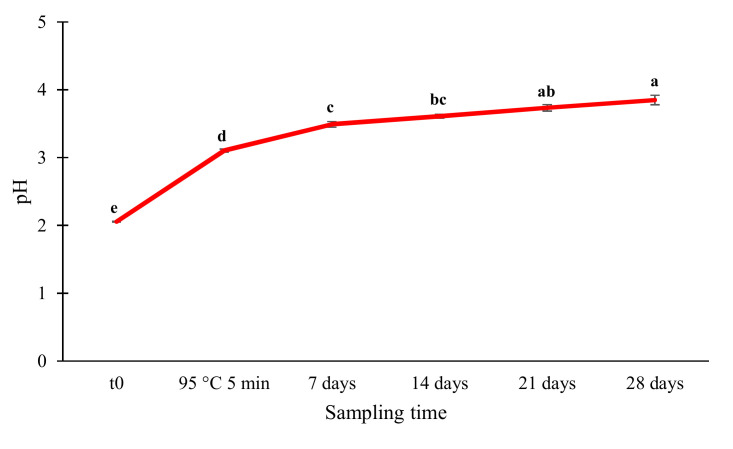
Results of pH measurements into acidified sea fennel preserves. Values are expressed as means ± standard deviation. For each sampling time, values labelled with different letters are significantly different (*p* < 0.05) by one-way ANOVA test.

As far as the acidification of brine is concerned, it reached an equilibrium pH of 3.85 ± 0.07 after 4 weeks of incubation at room temperature since the addition of 0.5% food grade lactic acid. The marked increase in the brine pH observed in the days after the addition of lactic acid (Figure 3) can be ascribed to the diffusion of this organic acid into the sea fennel tissues and hence to the previously reported buffer capacity of this herb [22,23].

Regarding probiotics, the commercially available formulation SYNBIO^®^ and the strain *L. plantarum* IMC 509 were separately inoculated into the sea fennel preserve.

SYNBIO^®^ has previously been exploited for the functionalization of various food products, including ripened cheese, salami, chocolate, and ice-cream, to reach a final probiotic load of approximately 10^9^ CFU/daily dose of *Lacticaseibacillus rhamnosus* IMC 501^®^ and *Lacticaseibacillus paracasei* IMC 502^®^ mixed 1:1 [24]. The effects of food enrichment with SYNBIO^®^ on bowl habits of healthy adults were also assessed with very promising results [25]. *L. plantarum* IMC 509 is currently commercialized, in association with *L. rhamnosus* IMC 501^®^ and *L. paracasei* IMC 502^®^, in a vaginal formulation [26]. To the authors knowledge, no trials have ever been carried out with *L. plantarum* IMC 509 to develop probiotic-enriched foods.

**Figure 4:**
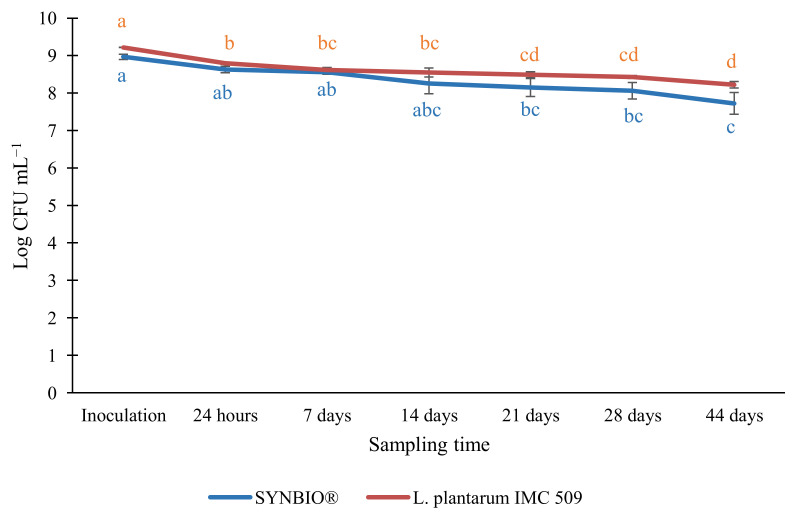
Results of the enumeration of *Lactiplantibacillus plantarum* IMC 509 (red line) and SYNBIO^®^ (blue line), a combination (1:1) of the strains *Lacticaseibacillus rhamnosus* IMC 501^®^ and *Lacticaseibacillus paracasei* IMC 502^®^), in an acidified sea fennel preserve, during a refrigerated storage of 44 days. Results are expressed as mean value of CFU mL^−1^ ± standard deviation. Values labelled with different letters in the same growth curve are significantly different (*p* < 0.05) by one-way ANOVA test.

*L. plantarum* IMC 509 and SYNBIO^®^ were inoculated into the acidified brine at a final load of 9.2 ± 0.0 and 9.0 ± 0.1 Log CFU mL^−1^, respectively. The enumeration of probiotics during prolonged storage at 4 ± 2 °C revealed a slight but continuous reduction of viable counts, which attested at 8.2 ± 0.1 and 7.7 ± 0.3 Log CFU mL^−1^ for *L. plantarum* IMC 509 and SYNBIO^®^, respectively, after 44 days (Figure 4).

This finding might be ascribed to the exposure of the inoculated microorganisms to stress conditions, such as the high concentration of sea fennel phenolic compounds and essential oils with an acknowledged antimicrobial activity [27,28,29] but mostly the low pH of the brine due to the addition of 0.5% of lactic acid to guarantee microbial safety and stability of the sea fennel preserve. In fact, although lactic acid is the major product of sugar fermentation and virtually acts as an antimicrobial agent against competing microorganisms, its accumulation, and hence the prolonged exposure to acidic conditions, usually results in death of lactic acid bacteria and, in particular, of probiotics [30].

**Figure 5:**
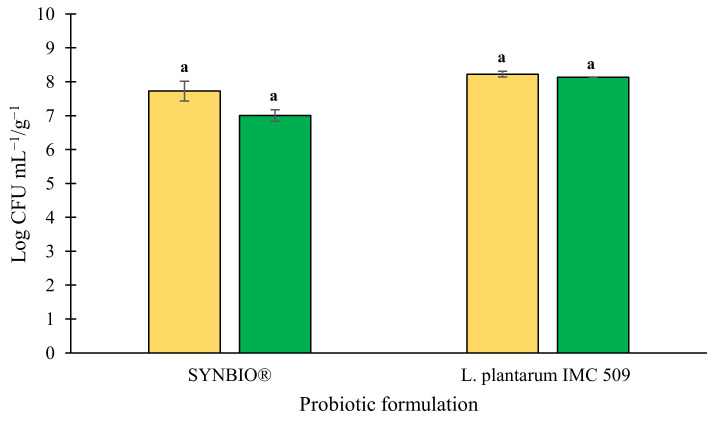
Loads of SYNBIO^®^ and *Lactiplantibacillus plantarum* IMC 509 in brine (yellow bar) and on drained (green bar) sea fennel leaves, assessed after 44 days storage under refrigeration. For each probiotic formulation, values labelled with different letters are significantly different (*p* < 0.05) by one-way ANOVA test.

Notwithstanding this, at the end of the monitoring period, the enumeration of probiotics adhering on drained sea fennel sprouts revealed bacterial loads comparable to those found in the brine salt solution (*p* > 0.05) and attesting at 8.1 ± 0.0 Log CFU g^−1^ for *L. plantarum* IMC 509 and 7.0 ± 0.2 Log CFU g^−1^ for SYNBIO^®^, respectively (Figure 5). Both these loads were comparable to quantities of probiotics in processed foods established as efficient for benefiting human health (10^6^–10^7^ CFU g^−1^) [31], and equivalent to 10^8^–10^9^ CFU of probiotics provided by a daily consumption of 100 g or 100 mL of probiotic-enriched foods [32].

This finding clearly suggests the occurrence, in the specific substrate assayed, of sea-fennel-derived nutrients able to support the survival of the inoculated probiotic strains, in agreement with what has previously been suggested for other probiotic-enriched foods [24]. Even the addition of 1% fructose to the brine salt solution might have had a beneficial impact since this carbohydrate has previously been reported to enhance the survival of probiotic lactobacilli [33]. In addition to this, the specific storage conditions applied, such as the use of glass jars hermetically sealed with stainless steel caps and refrigeration at 4 ± 2 °C, might also have contributed to the survival of the assayed probiotic strains at the right dose. In fact, glass has previously been reported as an ideal packaging material for probiotic-enriched foods, being characterized by an extremely low oxygen permeability that promotes the survival of probiotic lactic acid bacteria isolated from the human intestinal tract with a high sensitivity to high oxygen levels [5]. Even storage at 4–5 °C has previously been recommended to guarantee a prolonged viability of probiotic bacteria, which is inversely correlated to temperature [5].

## 4. Conclusions

The results overall collected in the present study demonstrated that artificially acidified sea fennel sprouts in brine can be an excellent vehicle to deliver probiotics to humans, given the high viability of both the probiotics assayed during storage under refrigeration. In more detail, for both *L. plantarum* IMC 509 and SYNBIO^®^, viable counts higher than the minimum loads suggested for health beneficial effects were found either in the brine or on drained sea fennel after 44 days of storage at 4 °C. On the one hand, these findings suggest the anchorage of the probiotic cells on the vegetable tissues of sea fennel, and on the other hand, the good adaptation of all the assayed probiotic strains to the sea fennel substrate. Hence, the consumption of 100 g per die of drained sea fennel sprouts soaked in acidified brine carrying more than 1 billion of *L. plantarum* IMC 509 or a mixture of *L. rhamnosus* IMC 501^®^ and *L. paracasei* IMC 502^®^ will guarantee the intake of 9 Log CFU g^−1^ of probiotics.

A great advantage of the probiotic-enriched preserve herein developed is that probiotic-enriched drained sea fennel can either be consumed as such or used as an ingredient for the preparation of other probiotic foods. A further advantage is that the intake of probiotic-enriched drained sea fennel provides a dose of probiotic bacteria comparable to that occurring in dairy-based probiotic foods, such as yogurt or fermented milks.

Moreover, differently from probiotic foods consisting of a liquid matrix, where probiotics are suspended in, when vegetables (such as sea fennel leaves and sprouts) are exploited as carriers of probiotics, the bacterial cells are immobilized and this guarantees the safe and effective transit of probiotic bacteria through the gastro-intestinal tract. In addition to this, the stable binding of probiotics to sea fennel tissues improves the bacterial resistance to the deleterious effects of gastric juices.

Further in vivo feeding trials are needed to assess the capability of the probiotic-enriched sea-fennel-based preserve of delivering viable bacteria into the human gastrointestinal tract.

## Data Availability

Data is contained within the article.
